# Editorial: Design and synthesis of natural antibacterial derivatives

**DOI:** 10.3389/fphar.2026.1837910

**Published:** 2026-04-23

**Authors:** Shikai Song, Ran Wei, Liyuan Li, Yupeng Zhang, Lele Wang, Yao Wang, Yunpeng Yi

**Affiliations:** 1 Shandong Provincial Key Laboratory of Livestock and Poultry Breeding, Institute of Poultry Science, Shandong Academy of Agricultural Science, Jinan, Shandong, China; 2 National Key Laboratory of Veterinary Public Health Safety, College of Veterinary Medicine, China Agricultural University, Beijing, China; 3 Shandong Animal Disease Prevention and Control Center (Shandong Provincial Zoonotic Disease Surveillance Center), Jinan, Shandong, China

**Keywords:** antibacterial derivatives, drug resistance, natural products, structural modification, synergistic combination

Antibiotic resistance is rising at an alarming rate, yet new antimicrobial agents remain scarce. Natural products offer exceptional structural diversity and inherent bioactivity, but their clinical translation is hindered by poor stability, bioavailability, and efficacy. Increasingly, researchers are addressing these limitations by using natural compounds as antibacterial leads, synergistic components, and modifiable scaffolds for structural optimization and advanced delivery. This integrated approach defines the progress highlighted in this Research Topic.

**FIGURE 1:**
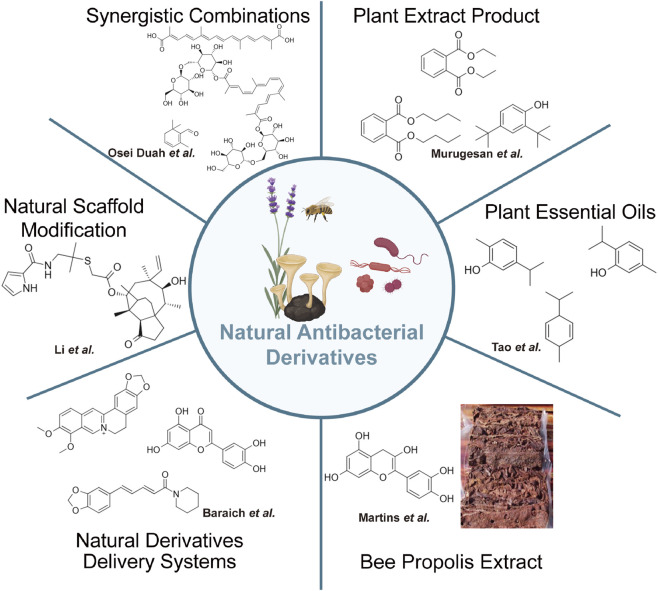
Key insights and research advances from the six contributions in this Research Topic, covering antibacterial activity, synergistic effects, structural optimization, and delivery system development of natural products for combating drug-resistant pathogens.

The six contributions in this Research Topic illustrate how natural products continue to serve not only as a source of antimicrobial leads but also as a foundation for subsequent optimization (Table 1; Figure 1). Several studies reaffirmed the intrinsic antibacterial activity of plant- and bee-derived compounds. Murugesan et al. identified 2,4-di-tert-butylphenol from *Clidemia hirta* as an active agent against *Pseudomonas aeruginosa*, while Tao et al. demonstrated that carvacrol, a major constituent of oregano essential oil, exhibits broad-spectrum bactericidal activity. Similarly, Martins et al. reported the potent antimycobacterial activity of Brazilian red propolis extract, and Osei Duah et al. identified diverse plant-derived scaffolds as promising candidates for treating ocular tuberculosis. Together, these findings reinforce the continued relevance of natural sources in antimicrobial discovery.

**TABLE 1:** Natural product-based antibacterial strategies and mechanisms.

Study	Natural agent	Partner drug/Agent	Target germ	Main mechanism
Osei Duah et al.	Safranal	Crocin, Crocetin	*E. coli* *S. aureus*	Multiple entry points into the germ
Tao et al.	Carvacrol	Tobramycin	MRSA, *E. coli*	Perforating the cell membrane
Li et al.	Pleuromutilin core	Pyrrole group	MRSA, MRSE	Blocking the PTC in the ribosome
Martins et al.	Red propolis	Biofilm, Macrophages	*M. tuberculosis*	Inhibiting biofilm and clearing intracellular bacilli
Murugesan et al.	Clidemia hirta	PBP2a protein	*P. aeruginosa*	Binding and inhibiting the cell wall protein

Beyond their inherent antibacterial activity, these studies further highlight the critical role of combination strategies in enhancing antimicrobial efficacy. Tao et al. showed that carvacrol can potentiate tobramycin activity via disruption of bacterial membrane integrity, while Baraich et al. employed a simplex-centroid mixture design to reveal pronounced synergistic interactions among saffron-derived compounds. These observations reflect a broader shift toward leveraging synergistic combinations to amplify antibacterial performance and address resistance.

At the same time, translating natural compounds into clinically viable agents increasingly depends on structural refinement and advanced delivery strategies. Osei Duah et al. emphasized the role of nanocarriers, liposomal systems, and *in situ* gels in overcoming pharmacokinetic and physiological barriers, particularly in ocular applications. Complementing this, Li et al. developed a pleuromutilin derivative with potent activity against multidrug-resistant Gram-positive bacteria, demonstrating how targeted structural modification can improve efficacy, safety, and resistance profiles.

Taken together, the studies featured in this Research Topic highlight a clear transition from simple activity screening toward more integrated approaches that combine discovery, synergistic optimization, and rational design. These strategies will likely play a central role in advancing natural products from promising bioactive compounds to clinically relevant antimicrobial agents.

Looking ahead, continued progress in this field will rely on close integration across disciplines, including chemistry, microbiology, materials science, and pharmacology. Multidisciplinary collaboration will be essential to fully realize the therapeutic potential of natural products and to develop effective and safe antimicrobial agents capable of addressing the ongoing challenge of drug resistance.

